# A Quantitative Measure of Electrostatic Perturbation in Holo and Apo Enzymes Induced by Structural Changes

**DOI:** 10.1371/journal.pone.0059352

**Published:** 2013-03-14

**Authors:** Sandeep Chakraborty

**Affiliations:** Department of Biological Sciences, Tata Institute of Fundamental Research, Mumbai, India; Wake Forest University, United States of America

## Abstract

Biological pathways are subject to subtle manipulations that achieve a wide range of functional variation in differing physiological niches. In many instances, changes in the structure of an enzyme on ligand binding germinate electrostatic perturbations that form the basis of its changed catalytic or transcriptional efficiency. Computational methods that seek to gain insights into the electrostatic changes in enzymes require expertise to setup and computing prowess. In the current work, we present a fast, easy and reliable methodology to compute electrostatic perturbations induced by ligand binding (MEPP). The theoretical foundation of MEPP is the conserved electrostatic potential difference (EPD) in cognate pairs of active site residues in proteins with the same functionality. Previously, this invariance has been used to unravel promiscuous serine protease and metallo-β-lactamase scaffolds in alkaline phosphatases. Given that a similarity in EPD is significant, we expect differences in the EPD to be significant too. MEPP identifies residues or domains that undergo significant electrostatic perturbations, and also enumerates residue pairs that undergo significant polarity change. The gain in a certain polarity of a residue with respect to neighboring residues, or the reversal of polarity between two residues might indicate a change in the preferred ligand. The methodology of MEPP has been demonstrated on several enzymes that employ varying mechanisms to perform their roles. For example, we have attributed the change in polarity in residue pairs to be responsible for the loss of metal ion binding in fructose 1,6-bisphosphatases, and corroborated the pre-organized state of the active site of the enzyme with respect to functionally relevant changes in electric fields in ketosteroid isomerases.

## Introduction

The modulation of the catalytic rates of enzymes is a critical aspect for the regulation of flux in biological pathways [1]. Since the structure of an enzyme is a key determinant of its functionality, this modulation is often achieved through the allosteric binding of various cofactors that restructure the active site [2]. An alternate strategy makes use of various effector molecules that bind to activator or repressor proteins, and influence their transcriptional efficiency through subtle structural changes [3], [4]. In many cases, the structural changes arising from mutations lead to depletion of metal ions, misfolding, aggregation and pathological phenotypes [5]. There are several such examples of sophisticated manipulations devised by evolution to effect a wide range of functional variation in differing physiological niches [5], [6].

We have established the non-triviality of the spatial and electrostatic congruence in cognate pairs of residues in the active site in several enzymes with the same catalytic function (CLASP) [7]. CLASP was used to unravel a serine protease scaffold in alkaline phosphatases [7], and a scaffold recognizing a -lactam (imipenem) in a cold-active *Vibrio* alkaline phosphatase [8], [9]. Since the correlation in the electrostatic potential difference (EPD) between residues is established, the comparison of EPD in two different structures of the same protein should indicate residues/domains of the protein that undergo significant deviation in electrostatic properties. These perturbed domains are likely to be the sites responsible for the different response of the protein to native and non-native substrates. CLASP analysis of the EPD in the catalytic residues in Class A β-lactamases identified the dichotomy in the proton abstraction mechanism [10], which led to a method for enumerating the possible pathways for proton abstraction in the active site [11].

Enzymes accelerate reactions by many orders of magnitude compared to the uncatalysed reaction in water [12], [13]. Although it has been long known that enzymes achieve these phenomenal rate accelerations by binding the transition state better than the substrate and thereby lower the activation energy, the exact roles of various factors (steric effects, conformational fluctuations, dynamical effects, electrostatic pre-organization, entropy changes, reactant destabilization, quantum tunneling, etc.) in accomplishing this is highly debated [14], [15]. Computational studies based on quantum, classical, and statistical techniques have evolved in the last few decades for studying the effect of structural changes on the chemical competency of the active site in enzymes. Typically these methods can access the free energy landscape for a chemical reaction through quantum mechanical (QM) calculations on model substrates [16], through molecular mechanical (MM) empirical functions [17]–[20] or through a hybrid quantum mechanical molecular mechanical (QM/MM) approach [21]. These computational approaches can be combined with accelerated conformational sampling methods (free energy perturbation [22], umbrella sampling [23], etc.) to finally obtain the free energy profile for a chemical reaction of interest. Ultrahigh-resolution diffraction studies [24]–[27] and photoelectron spectroscopy [28] are complementary experimental methods usually applied to gain insights in the critical residues involved in catalysis in enzymes. Active site residues have pK_a_ values that differ considerably from their intrinsic values [29], [30], and these deviations, computed using macroscopic [31], microscopic [32] or empirical models [33], have been used for predicting active site residues [34]–[36]. The pK_a_ values obtained from various methods have been recently benchmarked using experimental values, concluding that `the most striking result of this blind test was that nobody performed significantly better than the rest' [37]. A “highly parallelized molecular dynamics code on a high-performance capability computer" has revealed the initial stages of misfolding caused by metal depletion in Cu-Zn superoxide dismutase [38]. Another work was guided by computationally derived pK_a_ shifts to search for, and eventually detect, a tyrosinate in the active site of a bacterial ketosteroid isomerase [39]. These methods require considerable expertise, in terms of both understanding the computational algorithm and the understanding of the biological system, for setting up the simulations and can be computationally intensive. A fast, simple and accurate method to probe the electrostatic perturbations resulting from structural changes would be quite useful for such studies.

In the current work, we present a methodology for measuring electrostatic perturbation in proteins by contrasting the holoenzyme to the apoenzyme - **M**easuring **e**lectrostatic **p**erturbations in **p**roteins (MEPP).

MEPP takes as input two protein structures (mutations are accepted, but not insertions or deletions) and extracts the electrostatic potentials using APBS [40]. The perturbation of each residue with respect to its neighboring residues (within a parameterized distance) in the two structures is computed. In the default mode all amino acids are included, whereas in a stricter mode we consider only polar residues. Residue pairs that undergo polarity reversal are enumerated and Pymol scripts are generated for the visualization of the perturbed residues/domains.

The methodology of MEPP has been demonstrated on several enzymes that employ varying mechanisms to perform their roles. We have attributed the change in polarity in key residue pairs to be responsible for the loss of metal ion binding in some enzymes [2]. Further, some regulatory enzymes modulate their functionality by inducing structural changes in a domain (which undergoes little change in the electrostatic profile) using larger electrostatic perturbations in another domain on binding of cofactors [3], [4]. Some enzymes act through a motion induced domino effect in adjacent subunits, and have minimal electrostatic perturbations since structural changes suffice to produce the desired changes [6], [41]. We also corroborated the pre-organized state of the active site of the enzyme with respect to functionally relevant changes in electric fields in ketosteroid isomerase, a prototype enzyme for gaining insights into the electrostatics of catalytic reactions [42], [43]. Finally, certain pathological phenotypes resulting from mutations and metal-depletion are shown to undergo electrostatic perturbations in the critical residues [5], [38]. Thus, MEPP provides a fast, easy and reliable methodology to compute electrostatic perturbations induced by ligand binding, and has invariably identified relevant residues in all the enzymes we have studied.

## Results and Discussion

We demonstrate the application of the MEPP methodology on different enzymes. These enzymes use varying mechanisms to perform their respective roles. The choice of the radial distance that encompasses interacting residues needs to be made judiciously. A small radius will not include enough residues, while a large one will include irrelevant ones. The small runtime of MEPP allows fast iterations to help decide a reasonable value.

We have chosen a radial distance of 6Å to compute interacting residues in the examples shown hereafter, and have demonstrated the invariance of results based on the radius within reasonable limits.

### 1 Diphtheria toxin repressor

**Figure 1:**
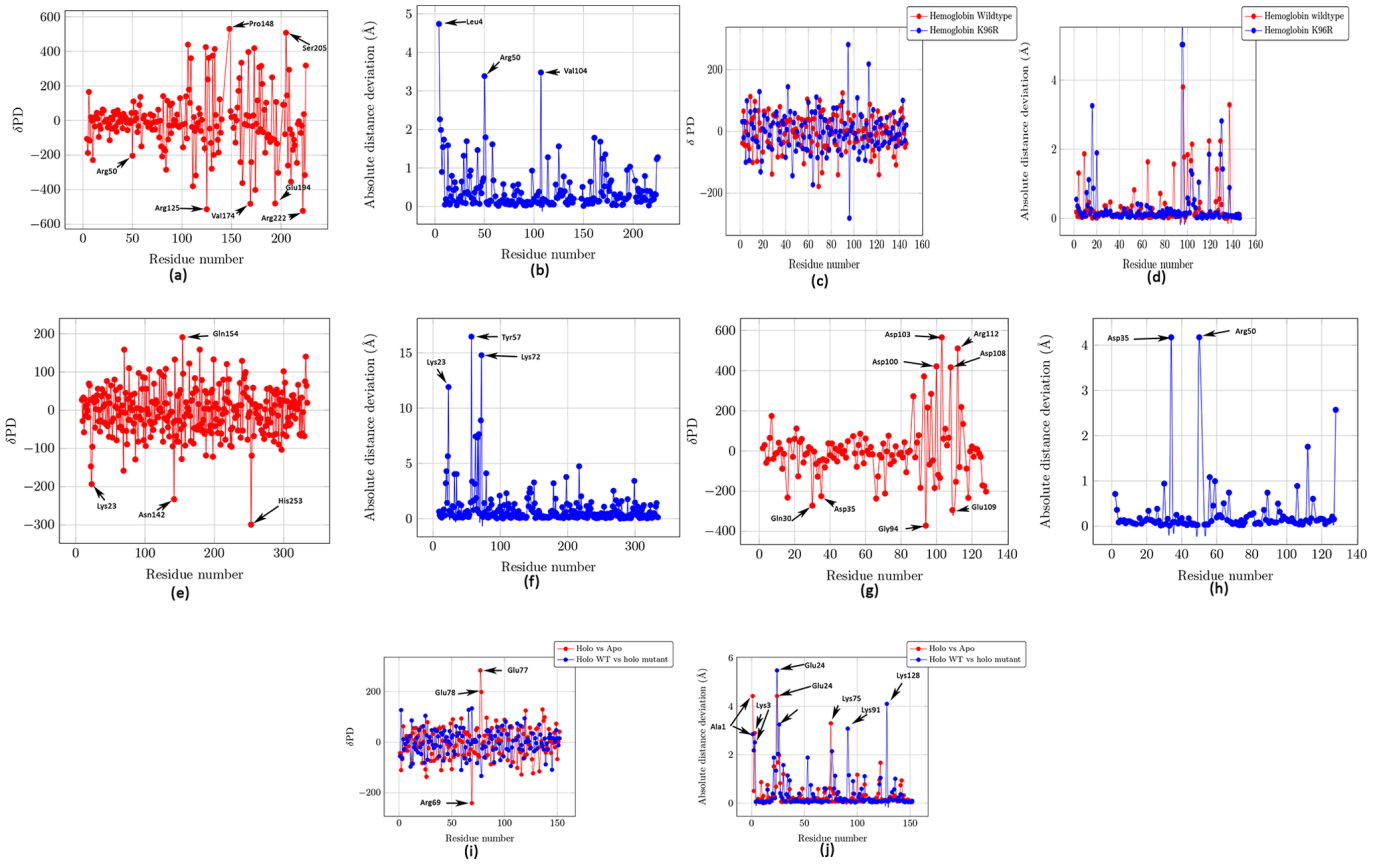
Electrostatic and spatial pertubations in different enzymes on ligand binding. Perturbations for each residue is computed with respect to residues within a radial distance of 6A. (a) and (b) Diphtheria toxin repressor (holoenzyme:1BI3/apoenzyme:1BI2), (c) and (d) Hemoglobin WT (holoen-zyme:3SDH/apoenzyme:4SDH) Hemoglobin K96R (holoenzyme:3UHI/apoenzyme:3UHK), (e) and (f) Fructose 1,6-bisphosphatase (holoenzyme:1EYK/apoenzyme:1CNQ), (g) and (h) Ketosteroid isomerase (holoenzyme:3IPT/apoenzyme:1OPY), (i) and (j) Cu-Zn superoxide dismutase WT (holoenzyme:1HL5/apoenzyme:1HL4), and mutant A4V (holoenzyme:1UXM/apoenzyme:1HL5)

The diphtheria toxin repressor (DtxR) is a metal ion-activated repressor found in Gram-positive prokaryotes [4], [44]. A 12- amino acid flexible tether links the N-terminal domain (residues 1–136) to the C-terminal. A mutation of either one of the residues in the primary metal binding site (Met10, Cys102, Glu105, or His106) to alanine results in complete inactivation of repressor activity [45]. We compared the holoenzyme (PDBid:1BI3) (which ligands a zinc ion) to the apoenzyme (PDBid:1BI2) of the DtxR from *Corynebacterium diphtheriae* [2]. We observed that the N-terminal undergoes minimal electrostatic perturbation (Fig. 1a), but larger conformational changes than the C-terminal (Fig. 1b), and the tether residues are where the residues having either low or large electrostatic perturbations are demarcated. Further, the significant polarity reversal in His106 and Glu105 is an explanation for their critical roles determined through mutational studies (Table 1) [45]. Other residue pairs with major polarity reversals are ones that bind the ancillary metal ion site (Glu83 and His79), and one of the residues that "contributes two ligands, Glu170 and Gln173, to the ancillary metal ion-binding site" (Gln173 and Asn169) [3] Lastly, the Arg50 present in the HTH region has been shown to be essential for nucleic acid binding [44], and is seen to have both structural and electrostatic perturbation (Fig. 1a and b).

**Table 1:** Residue pairs that undergo significant polarity reversal: PD1 and PD2 are the electrostatic potential difference between residues `a' and `b' in the holoenzyme and apoenzyme, respectively.

Apo PDB/Holo PDB	a	b	PD1	PD2	δPD
Diphtheria toxin repressor 1BI3/1BI2	GLU83	HIS79	8.6	–150.7	159.2
	ASP88	ARG84	70.6	–117.2	187.8
	HIS106	GLU105	435.8	–18.2	454
	ASP122	ARG84	19.7	–182.3	202
	GLN173	ASN169	571.5	–126.3	697.8
	GLU225	ARG114	284.5	–33.6	318.1
Hemoglobin WT 3SDH/4SDH	SER70	HIS69	77.8	–143.2	221
	ASP89	ASN86	73.9	–79.1	153
Hemoglobin K96R 3UHI/3UHK	GLU95	ARG96	47	–233.9	280.9
	THR103	ARG104	85.3	–75.9	161.1
Fructose 1,6-bisphosphatase 1EYK/1CNQ	GLU97	GLU98	95.6	–66.5	162.1
	GLN154	ASN142	44.5	–188.4	232.9
	TYR164	HIS253	217.3	–82.1	299.4
	ASN179	ARG198	19.9	–165.8	185.7
	TYR240	TYR226	61.3	–106.6	168
Ketosteroid isomerase 3IPT/1OPY	HIS110	GLU109	59.4	–94.2	153.6
Cu-Zn superoxide dismutase 1HL5/1HL4	GLU77	ARG69	15.3	–267.8	283.1
	HIS120	HIS48	27.7	–138.4	166.1

δPD  =  (PD1 - PD2). Electrostatic potential in dimensionless units of kT/e where k is Boltzmann's constant, T is the temperature in K and e is the charge of an electron. Threshold value for PD is 150 PD units.

**Figure 2:**
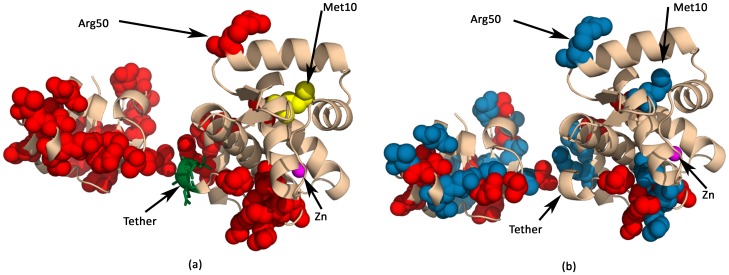
Visualizing the residues/domains that undergo significant electrostatic perturbation in the diphtheria toxin repressor (PDBid:1BI3). Threshold value above which perturbation are considered significant is 200 potential (difference) units. It can be seen that the C-terminal undergoes more electrostatic changes than the N-terminal. (a) Residues undergoing electrostatic perturbation of magnitude greater than the threshold value are colored in red and yellow (Met10). Met10 ligands metal ions, and its mutation to alanine results in complete inactivation of repressor activity. (b) Residues undergoing positive and negative electrostatic perturbations greater than the threshold value are colored in red and blue respectively. As expected, they are interspersed in order to maintain electrostatic homogeneity.

Fig. 2 shows the residues that have significant electrostatic perturbation, and clearly demonstrates the different responses of the N and C-terminals of the protein to the binding of the ligand. The Pymol script to visualize such perturbations is emitted during MEPP processing.

The N-terminal domain of DtxR that “undergoes a dynamic structural organization leading to homodimerization and target DNA binding" [45], but minor electrostatic changes can be explained by the fact that most repressors binds DNA in the apoenzyme loosely and intermittently. Hence, it is logical to assume that the electrostatics should be favorable to the configuration required for DNA binding. Electrostatic stimulation in the C-terminal transfers structural change to the N-terminal via a tether, enhancing the binding affinity. The invariance of electrostatic perturbation based on the radial distance from the active site used to choose interacting residues is seen in Fig. S1.

### 2 A Co-operative Dimeric Hemoglobin

Hemoglobins are proteins that transport oxygen in all vertebrates and in some invertebrates [6], [41]. The ligand affinity of each subunit is influenced by the conformation changes in the neighboring subunits on ligation [6]. The origins of the cooperativity in hemoglobin through quantitative calculations of the free energy have been previously done by force field calcuations [46] and by mathematically formulating the thermodynamic concept of order of free energy couplings [47], [48]. These computations are much more rigorous than those used by MEPP, and consequently allow a more detailed analysis of the cooperativity in these enzymes. We compared the holoenzyme of the homodimeric hemoglobin HbI from *Scapharca inaequivalvis* which has bound CO and heme (PDBid: 3SDH) to the apoenzyme which is CO free (PDBid: 4SDH) [41]. Table 1 shows that Ser70 and His69 has a major polarity reversal, which corroborates “the strategic location of the distal histidine" next to the subunit junction [6]. .

**Figure 3:**
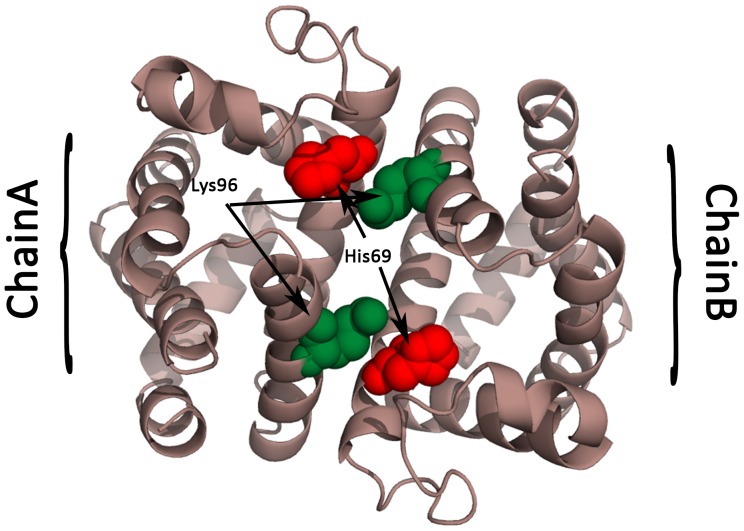
Residues involved in intersubunit coupling in hemoglobin (PDBid:3SDH). Lys96 (in green) in one subunit is in close proximity to His69 (in red) in the neighboring subunit. MEPP detects significant electrostatic perturbation in His69 and not in Lys96. On the other hand, Lys96 undergoes a distinct conformational change.

However, we do not see significant electrostatic perturbation around Lys96 and Phe97 (Fig. 1c), although there is a reasonable spatial rearrangement for Lys96 (Fig. 1d). These residues “cover the heme binding pocket in the ligated state and swing open in the unligated state„ [6]. The lack of electrostatic perturbation fits well with the proposed mechanism of cooperative ligand induced motion where there the dimer allows “slight relative rotation but preventing sliding„ [6]. Lys96 is involved in the intersubunit coupling. Fig. 3 shows the proximity of His69 to the Lys96 from the neighboring subunit, reaffirming the MEPP identification of His69 as a critical residue for the mechanism of catalysis in hemoglobin. Interestingly, when we compared the holoenzyme (bound CO and heme, PDBid: 3UHI) to the apoenzyme (CO free, PDBid: 3UHK) for the K96R mutant, we observe a distinct electrostatic perturbation for Arg96, a meaningful polarity reversal between Glu95 and Arg96, and a conformational change for Arg96 as well (Fig. 1d).

### 3 Fructose 1,6-bisphosphatase

Fructose 1,6-bisphosphatase is a critical regulatory enzyme in gluconeogenesis that requires divalent cations for activity, and is inhibited allosterically by adenosine monophosphate (AMP) [49]. We compared the holoenzyme (PDBid:1EYK) (which ligands AMP) to the apoenzyme (PDBid:1CNQ) [2]. In the apoenzyme, zinc ions binds to all three metal sites and magnesium ions to two of three metal sites, while in the holoenzyme the divalent cations bind to a single site [2]. The residues liganding metal in the apoenzymes are M1 = (Glu97, Asp118, Leu120), M2 = (Asp118, Asp121, Glu280) and M3 = (Asp68, Glu97), while in the holoenzyme the single metal is liganded by residues (M2 = Asp118, Asp121, Gly28) (metal site numbering is non-standard). Table 1 shows the pairs of residues that have a significant reversal in their potential differences in the two structures, which includes the pair (Glu97-Glu98). Thus, it is seen that the reversal of the potential in Glu97, which ligands two metal ions, has resulted in its rejection of the metal ions in metal binding sites M1 and M3.

Another residue pair that is observed to have undergone polarity reversal is Gln154 and Asn142. Once again, Asn142 is known to be an important residues as the “major AMP site interacts with Asn142 from an adjacent tetramer in the crystal, while the minor site does not" [49]. Fig. 1e also shows that Asn142 gains significant negative potential in the holoenzyme when compared to neighboring residues in the apoenzyme. However, we did not find any reference in existing literature to the critical nature of His253 which is indicated to have both a polarity reversal and accrual of potential (Fig. 1f and Table 1). Fig. 1f shows that Tyr57 and Ly72 undergo a significant spatial rearrangement, a fact that corroborates previous studies where fluorescence from a tryptophan Tyr57 mutation showed an altered conformational state for the loop 52–72 which plays a critical role in the mechanism of catalysis and allosteric inhibition of fructose 1,6-bisphosphatase [2].

### 4 Ketosteroid isomerase

Ketosteroid isomerase (KSI) catalyzes an allylic isomerization reaction at a diffusion-controlled rate, and is a prototype enzyme for gaining insights into the electrostatics of catalytic reactions [39], [42], [43], [50]. The relative importance of the electrostatic preorganization in the active site to achieve the phenomenal acceleration is highly debated. While some groups, proponents of the electrostatic preorganization theory [46], emphasize that `enzymes work by electrostatic preorganization' [42], [51], others have claimed that KSI `achieves its catalytic prowess through a combination of modest contributions from several mechanisms rather than from a single dominant contribution' [50]. We compared the holoenzyme (PDBid:3IPT,Y16S,D40N mutant) (which ligands equilenin, an intermediate analog) to the apoenzyme (PDBid:1OPY) from *Pseudomonas putida*. The mutation is intended to mimic the protonated D40 present in the intermediate complex. Fig. 1g shows that the active site undergoes minimal electrostatic perturbation. This corroborates the conclusion arrived by Jha et al. that the active site of the enzyme is pre-organized with respect to functionally relevant changes in electric fields since dipoles in the active site remained immobile with respect to the changing electric field [43].

### 5 Cu-Zn superoxide dismutase

**Figure 4:**
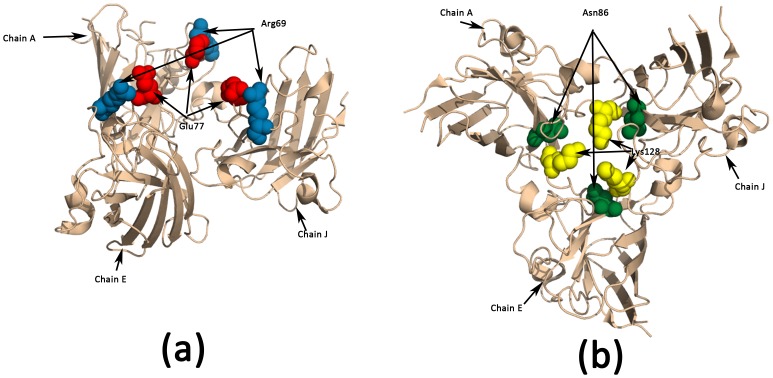
Residues involved in intersubunit interactions in Cu-Zn superoxide dismutase (PDBid:1HL5). (a) Glu77 (in red) and Arg69 (in blue) undergo significant perturbation. It can be seen that Glu77, which gains positive potential with respect to residues in the vicinity, is physically close to Glu77 from other subunits, and possibly generates a repulsive force that destabilizes the structure. (b) Asn86 (in green) and Lys128 (in yellow) from neighboring subunits are seen to be closely placed to each other, and are involved in H-bonds. Conformational changes in Lys128 possibly disrupt these bonds.

The mutant form of Cu-Zn superoxide dismutase (SOD1) is implicated in the hereditary form of the neurodegenerative disease Amyotrophic Lateral Sclerosis (ALS), commonly known as Lou Gehrig's disease, that effects neurons in the brain and spinal cord [52]. It has been shown that the metal-depleted SOD1 dimer gains toxic characteristics due to misfolding, unfolding, and aggregation [38]. We compared the holoenzyme (PDBid:1HL5, binds both Cu and Zn) to the apoenzyme(PDBid:1HL4, binds only Zn). The residues binding Zn are His63, His71, His80 and Asp83, while Cu is liganded by His48, His63, and His120. It is seen from Table 1 that His48 has a change in polarity with respect to His120. The spatial movement in this pair in the native structure has been previously noted [5]. Further, the other pair showing polarity reversal (Arg69-Glu77) was established as one of the 16 pairs that have long-range interactions with large interaction energies [52]. Also, Glu77 is a part of a non-native helix (Gly73-Glu77) that is adjacent to the zinc active site [53]. Fig. 4a shows that Glu77, which gains positive potential with respect to residues in the vicinity, is physically close to Glu77 from other subunits, and possibly generates a repulsive force that destabilizes the structure.

Next, we compared two holoenzymes (PDBid:1HL5 and 1UXM), where one of the holoenzymes (PDBid: 1UXM) has the mutation A4V [53]. This mutation has been implicated in generating a toxic phenotype. While Fig. 1i shows that there is little difference in the electrostatics in the protein, there are larger structural differences, especially at one of the electrostatic loop residues (Lys128) (Fig. 1j). In the apo enzyme, Asn86 is involved in H-bonds to residues Lys128-Gly130 of an adjacent SOD1 molecule [5], [38]. A conformational change in Lys128 possibly disrupts these bonds between the closely placed Asn86 and Lys128 in neighboring subunits (Fig. 4b).

### 6 Limitations

The inability of simple continuum electrostatic models, such as those used MEPP, to always give a true representation of the electrostatic milieu of the active site is a well-known fact [54], [55]. It has been argued that the electrostatic energies in proteins computed using such implicit models are sensitive to the value assigned to the dielectric constant [56]. However, MEPP computes the difference in electrostatic potentials rather than absolute values, and the (empirical) significance of the electrostatic potential difference in the active site has been established through several examples [7], [9], [11]. For example, although computational pK_a_ predictions for Tyr residues that form an extended hydrogen bond network in the oxyanion hole of the ketosteroid isomerase give a value more than 11, experimental results show the presence of tyrosinate in the active site [39]. Computations assuming the ionization of these Tyr residues were able to better explain the observed frequency shifts than those obtained by presupposing the ionization of Asp103, substantiating the initial error in estimating the pK_a_ values of the Tyr residues [39].

In order to obtain the most accurate values for electrostatic energies, one has to solve the Schrödingers equation for the wavefunction of the interacting atoms [57]. Due to interactions between electrons, this equation does not have analytic solutions for atoms with more than one electron [58]. *Ab initio* methods use various approximations, for example the orbital approximation which assumes a linear combination of the wavefunctions of each electron, to obtain solutions for multi-electron atoms. One such technique, the Hartree-Fock self consistent field, starts with an approximate form of all orbitals and iteratively approaches convergence for the energies of the various orbitals [58]. However, these methods are computationally unfeasible even for moderately large systems. The QM/MM method has been proposed which uses quantum mechanical analysis for the reacting fragments, and classical analysis for the rest of the protein [21]. Such methods produce more authentic results, but require expertise and intensive computational resources. Such methods produce more authentic results, but require expertise and intensive computational resources, and are routinely used to gain insights into the significance of electrostatic effects in catalytic reactions [15], [59]. It is to be noted that the current work is incapable of assigning the effects of the measured electrostatic perturbation.

The proper choice of the radial distance is another heuristic that needs to be varied keeping in the mind the differently shaped configurations of active sites in enzymes. The small runtimes of MEPP facilitates the iteration needed to derive such values. The variation of the perturbations in residues has been observed to be minimal (Fig. S1).

### 7 Summary

The theoretical foundation of the current work lies in the conserved electrostatic potential difference (EPD) in cognate pairs of active site residues in proteins with the same functionality. This similarity is observed in structures solved independently over many years, and also holds true for convergently evolved proteins (for example, in the two major families of serine proteases, chymotrypsin and subtilisin [60]). This also speaks highly of the reliability of the APBS and PDB2PQR implementations [40], [61].

Given that the EPD is significant, we expect differences in the EPD to be significant too. The EPD changes of one residue in two structures of the same protein with respect to residues in the vicinity should therefore indicate regions of the proteins that undergo significant electrostatic perturbation. The conformational changes accompanying the binding of a cofactor do not always imply a concomitant change in the electric field of significance. For instance, we found little electrostatic perturbation in hemoglobin, where the proposed model suggests that binding of the first ligand in one subunits induces a binding event in the other partner subunit [6].

Also, the electrostatic perturbation is not always uniform throughout the protein. The magnitude and location of these changes are primarily dictated by the mechanism through which the enzyme performs it role. For example, the N-terminal of the diphtheria toxin repressor (DtxR) undergoes minimal electrostatic perturbation in spite of large conformational changes. The situation is exactly reversed for the C-terminal. It is only logical that a regulatory enzyme, like the DtxR, should not undergo a dramatic change in its electrostatic profile, since such enzymes are still capable of binding substrate, albeit loosely, and intermittently perform their roles [3], [4]. This low affinity is due to structural considerations, and the enzyme using the electrostatics of an adjacent domain effects the required structural modifications. A definite reversal of polarity is desired in cases where the functionality changes require the gain/loss of a metal cofactor. Such is the case for fructose 1,6-bisphosphatase, where the binding of adenosine monophosphate results in the reversal of the polarity of residues that ligand two metal ions, resulting in the holoenzyme failing to bind these ions [2]. Thus, MEPP provides a fast, easy and reliable methodology to compute electrostatic perturbations induced by ligand binding, and has invariably identified relevant residues in all the enzyme we have probed.

## Methods

MEPP takes as an input two protein structures - the holo (P^h^) and the apo enzyme (P^a^). The number of residues in P^h^ and P^a^ are expected to be the same, although a (user defined) number of residue types are allowed to be different. In other words, mutations are accepted, but not insertions or deletions. APBS and PDB2PQR packages are used to calculate the potential of the atoms in the protein [40], [60]. Let us denote the potential of the reactive atom of residue R_i_ in protein P^x^ as Pot(P^x^,R_i_). The electrostatic potential difference (EPD) between two residues is given by Equation 1. For each residue Res_i_ in protein P^h^, we compute the set of residues (Φ(P ^h^)^neigh^_i_) that are within a parameterized radius P_rad_ (Equation 2).

Next, we compute the summation of the EPDs of residue R_i_ with respect to residues in Φ (P ^h^)^neigh^_i_ for both P^h^ and P^a^ (Equations 3 and 4). Note that for residues in the apoenzyme, we consider residues that are within radius P_rad_ in the holoenzyme (Φ (P ^h^)^neigh^_i_), although structural changes might have changed this set if it were to be computed on the apoenzyme. This ensures that the number of residues being compared is the same. Finally, we define the perturbation of residue R_i_ (E_i_^perturb^) as the difference in the summation of the EPDs of residue R_i_ with respect to residues in Φ (P ^h^)^neigh^_i_ (Equation 5).

MEPP also identifies the set of residue pairs that undergo a shift in polarity (Φ (x, y)^PolarityReverse^). This is computed by choosing residue pairs that have a change in sign in their EPD (magnitude of the product PD(P ^h^, Res_i_, Res_j_) * PD(P ^a^, Res_i_, Res_j_) should be less than zero), and the absolute magnitude of their

difference should be larger than a user defined threshold value (P_thresh_) (Equation 6).

An optional feature restricts E^perturb^ computations to polar residues only (Equation 7). MEPP outputs a Pymol script for the visualization of the perturbed residues/domains which highlights the residues that have undergone perturbations more than a user defined threshold. The colored regions can be further differentiated based on the polarity of the perturbation. An analysis similar to the one done for pairwise EPD is done for pairwise distance deviations. More sophisticated methods exist for quantifying spatial changes [62].

The MEPP package is written in Perl on Ubuntu. Hardware requirements are modest - all results here are from a simple workstation (2GB ram) and runtimes were a few minutes at the most. The APBS parameters were set as described previously in [7]. APBS writes out the electrostatic potential in dimensionless units of kT/e where k is Boltzmann's constant, T is the temperature in K and e is the charge of an electron. We extensively integrated and used the freely available BioPerl [63] modules and Emboss [64] tools. All protein structures were rendered by PyMol (http://www.pymol.org/). The source code is made available at www.sanchak.com/mepp.
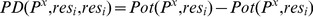
(1)

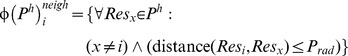
(2)

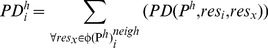
(3)

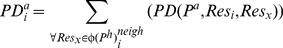
(4)

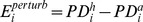
(5)

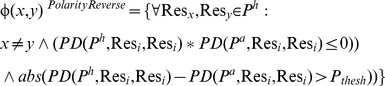
(6)

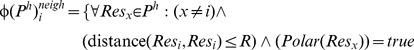
(7)


## Supporting Information

Figure S1 **Invariance of electrostatic perturbation based on radial distance from the active site used to choose interacting residues.** In the diphtheria toxin repressor from *Corynebacterium diphtheriae*, the C-terminal undergoes more electrostatic perturbation compared to the N-terminal, and this change is independent of the radial distance which defines interacting residues
(PDF)
