# Supplementary material for: A Quantitative Measure of Electrostatic Perturbation in Holo and Apo Enzymes Induced by Structural Changes
Source: PLoS One. 2013 Mar 14;8(3):e59352. doi: 10.1371/journal.pone.0059352 (PMC3597595; doi:10.1371/journal.pone.0059352)
Supplement: Figure S1 — Invariance of electrostatic perturbation based on radial distance from the active site used to choose interacting residues. In the diphtheria toxin repressor from Corynebacterium diphtheriae, the C-terminal undergoes more electrostatic perturbation compared to the N-terminal, and this change is independent of the radial distance which defines interacting residues (PDF) [file pone.0059352.s001.pdf]

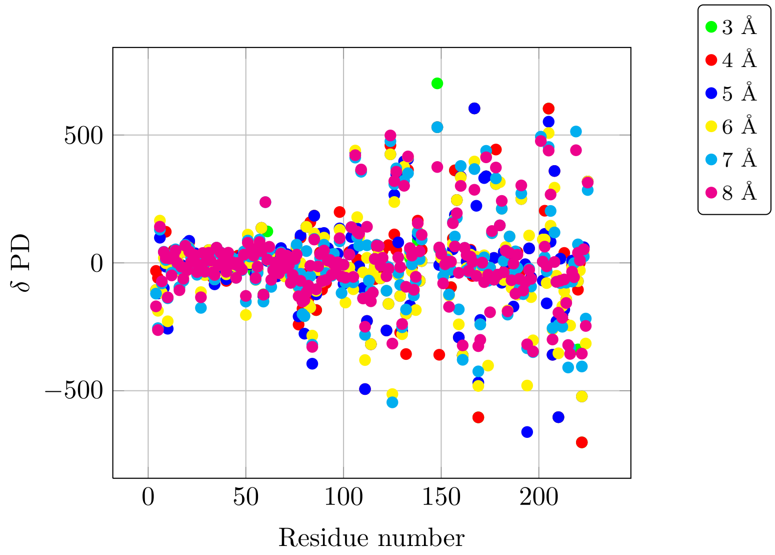

(a)

Fig. S1: **Invariance of electrostatic perturbation based on radial distance from active site used to choose interacting residues**: In the diphtheria toxin repressor from *Corynebacterium diphtheriae*, the C-terminal undergoes more electrostatic perturbation compared to the N-terminal, and this change is independent of the radial distance which defines interacting residues.
